# MB109 as bioactive human bone morphogenetic protein-9 refolded and purified from *E. coli* inclusion bodies

**DOI:** 10.1186/1475-2859-13-29

**Published:** 2014-02-24

**Authors:** Mario Meng-Chiang Kuo, Phuong Hong Nguyen, Yun-Hui Jeon, Subin Kim, So-Mi Yoon, Senyon Choe

**Affiliations:** 1 Protein Engineering Laboratory, joint Center for Biosciences, Songdo Smart Valley, 214 Sondgo-dong, Yeonsu-gu, Incheon 406-840, Korea; 2 Drug Discovery Collaboratory, Carlsbad Center for Translational Medicine, Carlsbad, CA 92008, USA

**Keywords:** TGF-beta, BMP-9, MB109, Refolding, Rapid dilution, *E. coli*, Recombinant cytokine

## Abstract

**Background:**

The development of chemical refolding of transforming growth factor-beta (TGF-β) superfamily ligands has been instrumental to produce the recombinant proteins for biochemical studies and exploring the potential of protein therapeutics. The osteogenic human bone morphogenetic protein-2 (hBMP-2) and its *Drosophila* DPP homolog were the early successful cases of refolding into functional form. Despite the similarity in their three dimensional structure and amino acid sequences, several other TGF-β superfamily ligands could not be refolded readily by the same methods.

**Results:**

Here, we report a comprehensive study on the variables of a rapid-dilution refolding method, including the concentrations of protein, salt, detergent and redox agents, pH, refolding duration and the presence of aggregation suppressors and host-cell contaminants, in order to identify the optimal condition to refold human BMP-9 (hBMP-9). To produce a recombinant form of hBMP-9 in *E. coli* cells*,* a synthetic codon-optimized gene was designed to encode the mature domain of hBMP-9 (Ser320 – Arg429) directly behind the first methionine, which we herein referred to as MB109. An effective purification scheme was also developed to purify the refolded MB109 to homogeneity with a final yield of 7.8 mg from 100 mg of chromatography-purified inclusion bodies as a starting material. The chemically refolded MB109 binds to ALK1, ActRIIb and BMPRII receptors with relatively high affinity as compared to other Type I and Type II receptors based on surface plasmon resonance analysis. Smad1-dependent luciferase assay in C2C12 cells shows that the MB109 has an EC_50_ of 0.61 ng/mL (25 pM), which is nearly the same as hBMP-9.

**Conclusion:**

MB109 is prone to be refolded as non-functional dimer and higher order multimers in most of the conditions tested, but bioactive MB109 dimer can be refolded with high efficiency in a narrow window, which is strongly dependent on the pH, refolding duration, the presence of aggregation suppressors and the concentrations of protein, salt and detegent. These results add to the current understanding of producing recombinant TGF-β superfamily ligands in the microbial *E. coli* system. An application of the technique to produce a large number of synthetic TGF-β chimeras for activity screen is also discussed.

## Background

Bone morphogenetic proteins (BMPs) are extracellular growth factors that belong to the transforming growth factor-beta (TGF-β) superfamily. Among the seventeen members in the BMP subfamily, BMP-9 is distinguished from other BMPs by its unique receptor-binding specificity and its diverse roles in a variety of cellular processes. For example, BMP-9, as well as BMP-10, signals only through the activin receptor-like kinase 1 (ALK1) to trigger downstream cellular responses, while the other BMP ligands interact promiscuously with multiple, different type I receptors [1-3]. BMP-9 can induce apoptosis in prostate cancer cells [4], promote the proliferation of ovarian cancer cells [5] and stimulate epithelia to mesenchymal transition in liver cancer cells [6]. It regulates the growth and migration of endothelial cells, thus also playing an essential role in angiogenesis [7,8]. BMP-9 is one of the most potent BMPs to induce osteogenic differentiation and orthotopic bone formation [9,10]. It also promotes chondrogenic differentiation in cultured multipotential mesenchymal cells and articular chondrocytes [11,12]. Due to these characteristics, BMP-9 has been suggested as an effective bone regeneration and tissue repair agent for clinical applications [13]. In addition to angiogenesis, osteogenesis and chondrogenesis, BMP-9 is also a potent inducer of the cholinergic phenotype in the central nervous system, and thus has the potential to be used in regenerative medicine to treat disease related to cholinergic neurons [14]. BMP-9 is additionally able to inhibit the production of hepatic glucose and to activate the expression of several key enzymes in lipid metabolism [15], which makes it a potential candidate for diabetes treatment and weight control.

Because of the diverse biological functions and pharmaceutical potentials, there is significant interest in the production of recombinant human BMP-9 (hBMP-9). A full-length hBMP-9 polypeptide contains 429 amino acid residues, including an amino-terminal signal peptide, a pro-region and a carboxyl-terminal mature domain. The bioactive hBMP-9 ligand is a disulfide-linked homodimer of the mature domain, from Ser320 to Arg429 (110 amino acids). Each polypeptide chain of the homodimer has seven cysteine residues, which form three intra-molecular and one inter-molecular disulfide bonds. As revealed in the X-ray structure, these seven disulfide bonds constitute the characteristic cystine knot and common structural scaffold of the TGF-β superfamily ligands [1]. Because of the complexity of this disulfide-linked molecule, production of sufficient amount of recombinant hBMP-9 ligand for research purposes had only been achieved with mammalian Chinese hamster ovary (CHO) cells [1,3,15]. The CHO cell expression system utilizes the innate mammalian cellular machinery to fold the molecule and form the disulfide bonds. After post-translational modifications, bioactive hBMP-9 is secreted into the conditioned medium and can be purified with chromatographic methods [16]. The CHO-derived hBMP-9 mature domain has a molecular weight close to its theoretic value on SDS-PAGE, which indicates that it is not glycosylated [1,15,16].

Although the CHO expression system allows the production and purification of sub-milligram to milligram quantities of bioactive BMPs directly from a liter of conditioned medium [17], it is costly and time consuming to establish a high-expressing, stable cell line. This disadvantage becomes a major technical barrier when tens or hundreds of chimera TGF-β ligands need to be generated simultaneously for activity screens [18]. Alternatively, a microbial system using *E. coli* cells has been developed to produce recombinant TGF-β superfamily ligands in a simple and economical manner. The osteogenic human bone morphogenetic protein-2 (hBMP-2) and its *Drosophila* DPP homolog were the early successful cases [19,20]. When overexpressed in *E. coli* cells, the mature domains of hBMP-2 and *Drosophila* DPP form water-insoluble inclusion bodies and require in vitro denaturation and renaturation to form their native protein conformations [19-22]. Common refolding variables and parameters derived from these early studies have been used as standard refolding conditions to successfully produce other BMP ligands, such as hBMP-3, hBMP-6, hBMP-2/6 heterodimer, hBMP-12, hBMP-13 and activin-BMP-2 chimeras with a yield sufficient for structural and functional studies [18,23-25]. However, the standard refolding conditions that work for the mentioned ligands are not generally applicable to refold other TGF-β superfamily ligands, even though these ligands share a common structural scaffold [18]. The exact reasons remain elusive for why the standard refolding conditions can only refold a limited numbers of TGF-β superfamily ligands.

In this study, we used *E. coli* cells as an expression host and created a synthetic, codon-optimized gene that encodes the mature domain of hBMP-9 (Ser320 – Arg429) directly behind a Met start codon, which we herein refer to as MB109. We did a comprehensive analysis of several variables to the standard refolding conditions to identify and optimize the critical variables and parameters to refold bioactive MB109 with high efficiency. An effective purification scheme was also developed to purify the refolded MB109 to homogeneity. Finally, the biological activity was confirmed by Smad1-dependent luciferase reporter assay and receptor-binding specificity performed with surface plasmon resonance analysis with immobilized extracellular domains of Type I and Type II receptors.

## Results

### Expression and purification of denatured, monomeric MB109

**Figure 1:**
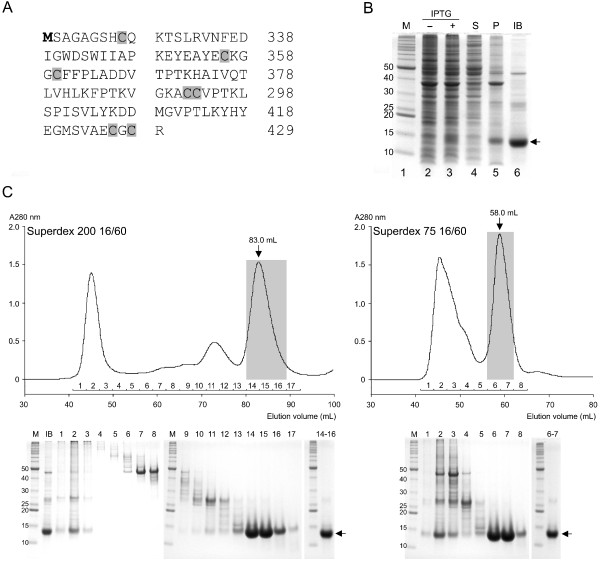
**Expression of MB109 in *E. coli *cells and purification of the isolated inclusion bodies. (A)** Amino acid sequence of the MB109 polypeptide expressed in this study. The first methionine (bold) is engineered to serve as translational start. The seven conserved cysteine residues are highlighted in gray. **(B)** Reduced SDS-PAGE gel of the expressed MB109 protein. Lane 2 and 3 are whole cell lysates before and after IPTG induction, respectively. Lane 4 and 5 are supernatant and pellet, respectively, of the IPTG-induced cell lysate. Lane 6 is isolated inclusion bodies. The black arrow indicates the monomeric MB109 protein. **(C)** Size exclusion chromatographic profiles of Superdex 200 16/60 (left-upper panel) and Superdex 75 16/60 (right-upper panel) loaded with the isolated inclusion bodies. Bottom panels are reduced SDS-PAGE gel images of each corresponding elution fractions. Gray bars highlight the fractions containing MB109 monomers. The black arrows indicate the monomeric MB109 protein.

To express the mature domain of hBMP-9, a synthetic, codon-optimized gene, encoding Ser320–Arg429 of NCBI Gene ID:2658 was cloned into a pET21a vector behind T7 promoter (Figure 1A). This recombinant gene product, which we refer to as MB109, contains Met as the N-terminus followed by the coding region of the mature domain of hBMP9. The expression plasmid was transformed into BL21 *E. coli* cells and the transformants were cultured in LB-broth under aerobic condition in a shaking incubator. After IPTG induction for 20 hours at 37°C, the cells were lysed by microfluidization and the expression of the target protein was analyzed by SDS-PAGE. In the whole cell lysate, MB109 was present in the non-soluble fraction and could be isolated by centrifugation, indicating the formation of inclusion bodies (Figure 1B, lane 5). About 150 mg inclusion bodies were isolated from one liter of cell culture.

**Figure 2:**
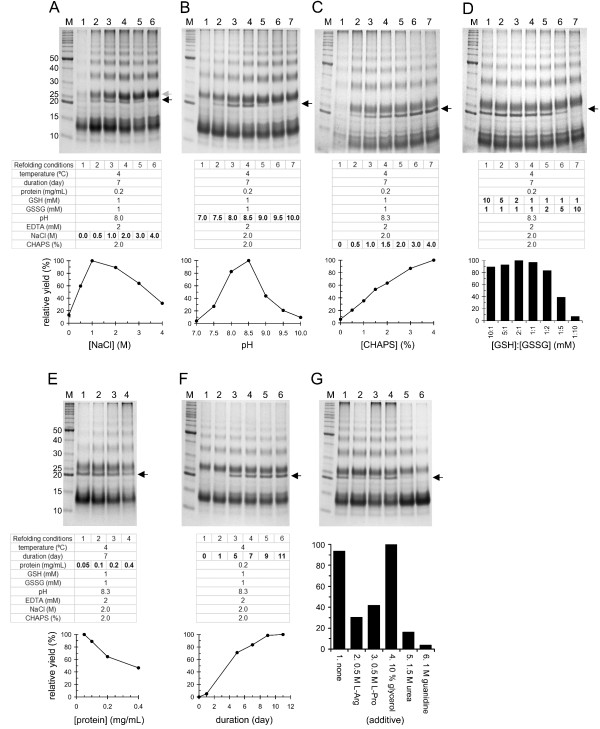
**Analysis of refolding variables to identify the optimal refolding conditions for MB109. (A)** Top panel is non-reduced SDS-PAGE gel image showing the result of refolding in 0–4 M NaCl. Black and gray arrows indicate the refolded functional and chemical dimers, respectively. Middle panel shows the detail of each refolding condition. Bottom panel is densitometry of the functional dimer bands in the top SDS-PAGE image. The data show relative refolding yield in each tested condition as compared to the highest one (set as 100%). **(B-F)** Refolding of MB109 at pH 7–10 **(B)**, in 0–4% CHAPS detergent **(C)**, in different molar ratios of reduced (GSH) and oxidized (GSSG) glutathiones **(D)**, at 0.05–0.4 mg/mL protein concentrations **(E)**, and the time course of MB109 Refolding at 4°C **(F)**. **(G)** Effect of secondary additives on the refolding of MB109. The background conditions were 1.5 mM NaCl, pH 8.3, 2% CHAPS, 1/1 mM GSH/GSSG, 0.2 mg/mL protein, 2 mM EDTA and 4°C for 7 days.

After washing three times, the inclusion bodies remained associated with some contaminant proteins (Figure 1B, lane 6). To further improve the purity of the MB109 protein prior to refolding screen, the isolated inclusion bodies were solubilized in reduced and denatured condition and subjected to chromatographic purification by size exclusion chromatography (SEC) using a HiLoad Superdex™ 75 or 200 column (GE Healthcare). We found that even though these two types of gel filtration columns have different separation ranges of molecular size, they gave similar results in terms of final purity and yield of the denatured monomeric MB109 protein. In the Superdex™ 200 column, the solubilized inclusion bodies were eluted as three major peaks at around 46, 72 and 83 mL, with the target MB109 protein present in the last peak (Figure 1C, gray bar). In the Superdex™ 75 column, the solubilized inclusion bodies were eluted as two, well-separated peaks, with the target MB109 protein present in the second peak (Figure 2D, gray bar). The fractions containing the monomeric MB109 protein were pooled and concentrated to 20 mg/mL for refolding. About 50 mg of monomeric MB109 was purified from 100 mg of solubilized inclusion bodies using either column.

### Effect of salt concentration on the refolding of MB109

The standard refolding conditions, derived from early studies to readily refold DPP and other BMPs, contain a minimal set of six components: detergent (CHAPS), salt (NaCl), chelator (EDTA), redox agents (reduced and oxidized glutathiones, GSH and GSSG, respectively) and buffer (Tris-HCl) [18,20,23-26]. To determine which variables are critical for refolding MB109, we first varied the NaCl concentration from 0 to 4 M and fixed the other refolding variables at their commonly used values (Figure 2A, middle panel). After 7 days incubation at 4°C, visible aggregates were spun down and the supernatants were concentrated to directly visualize the refolding results in non-reduced SDS-PAGE. In the absence of NaCl, most protein aggregated in the refolding solution and thus limited protein remained in the supernatant after centrifugation (Figure 2A, top panel, lane 1). In the presence of NaCl, MB109 refolded into monomer, dimer and higher order multimers (Figure 2A, top panel, lanes 2-6).

In the top panel of Figure 2A, there are two well-separated bands in Lanes 2-6 at around 20–30 kDa, which have a size close to the theoretic molecular weight of dimeric MB109 (24.4 kDa). The protein of the bottom band (black arrow) was purified and its bioactivity was subsequently confirmed (see below). We refer to it as “functional dimer” and we used it as a reference of correctly refolded MB109 on SDS-PAGE. The upper band (gray arrow), which is not bioactive after being purified (see below), is referred to as “chemical dimer.” The densitometry of the functional dimer bands in the SDS-PAGE image (Figure 2A, bottom panel) revealed that the optimal refolding salt concentration for MB109 was around 1.0–2.0 M. At salt concentrations increasingly above 2 M, the refolding efficiency was reduced gradually.

### Effect of pH on the refolding of MB109

To analyze the pH effect on refolding MB109, the salt concentration was fixed at 2 M and the buffer pH was varied between 7.0 and 10.0 at a 0.5 interval. As shown in the upper panel of Figure 2B, the functional dimer band was only observed between pH 7.5 and 9.5 (lanes 2-6). The chemical dimer, as well as monomer and multimer, were present in all tested pH conditions (lanes 1-7). The refolding efficiency of the functional dimer had sharp pH dependency. The optimal refolding pH was found to be between 8.0 and 8.5, and the refolding efficiency reduced drastically outside of this range (Figure 2B, bottom panel).

### Effect of detergent concentration on the refolding of MB109

In the standard refolding conditions, 1.8–2% CHAPS had been used to refold DPP and other BMPs [18,20,23-26]. To test the effect of CHAPS concentration on refolding MB109, 0 to 4% CHAPS was used in the refolding solution. In the absence of the detergent, MB109 formed visible aggregates in the refolding solution after 7 days of incubation, and thus little protein remained in the supernatant after centrifugation (Figure 2C, top panel, lane 1). At a CHAPS concentration above 0.5%, the functional dimer could be refolded; the refolding efficiency was positively correlated with the amount of CHAPS in the refolding solution (Figure 2C, bottom panel). When compared to the commonly used 2% CHAPS concentration, the yield of the functional dimer increased about 37% and 58% in the presence of 3% and 4% CHAPS, respectively.

### Effect of redox condition on the refolding of MB109

Because a bioactive BMP molecule contains several disulfide bonds, reduced and oxidized glutathiones (GSH and GSSG, respectively) had been used as a redox system in refolding solution to allow the formation and reshuffling of disulfide bonds. To identify the optimal redox condition, millimolar ratios between 10:1 and 1:10 of GSH and GSSG were tested (Figure 2D). Interestingly, the refolding efficiency of the functional dimer depended mostly upon the amount of GSSG, but not GSH, in the refolding solution. In other words, the more oxidizing “power” (GSSG) in the solution, the functional dimer was refolded less efficiently (Figure 2D, conditions 4-7). In contrast, increasing the reducing “power” (GSH) in the refolding solution did not significantly affect the refolding efficiency (Figure 2D, conditions 1-4). The maximal refolding efficiency was observed at the combination of 2 mM GSH and 1 mM GSSG.

### Effect of protein concentration on the refolding of MB109

In all of the refolding conditions tested above, a chemical dimer and higher order multimers of MB109 were prone to form over the functional dimer. This result suggests that the protein concentration (0.2 mg/mL) used for the refolding tests may be too high to form the functional dimer and instead cause the non-specific multimerization observed. To understand the effect of protein concentration on the refolding yield, protein concentrations between 0.05 and 0.4 mg/mL were tested (Figure 2E). Indeed, the refolding yield was reversely correlated with the protein concentration in the refolding solution. At 0.05 mg/mL, the tendency to form higher order multimers was significantly reduced (Figure 2E, top panel, lane 1), and the yield of the functional dimer increased about 55% as compared to the solution refolded at 0.2 mg/mL (bottom panel).

### Effect of refolding duration on the refolding of MB109

In addition to the chemical variables tested above, the refolding time course was analyzed for up to 11 days at 4°C to identify the time required to achieve maximal refolding yield. As shown in Figure 2F, the functional dimer band could be seen to after 2 days and the yield plateaued after 9 days of incubation.

### Effect of secondary additive on the refolding of MB109

Some chemical additives are known to be effective aids for *in vitro* protein refolding [27,28]. To determine whether the refolding yield could be further improved, we tested the commonly used aggregation suppressors and denaturants, including L-arginine, L-proline, glycerol, urea, and guanidine. As shown in Figure 2G, addition of 0.5 M L-arginine, 0.5 M L-proline, 1.5 M urea, and 1 M guanidine significantly reduced the efficiency of refolding the functional dimer, whereas the addition of 10% glycerol provided a minimal increase on the refolding yield.

### Effect of host cell contaminants on the refolding of MB109

**Figure 3:**
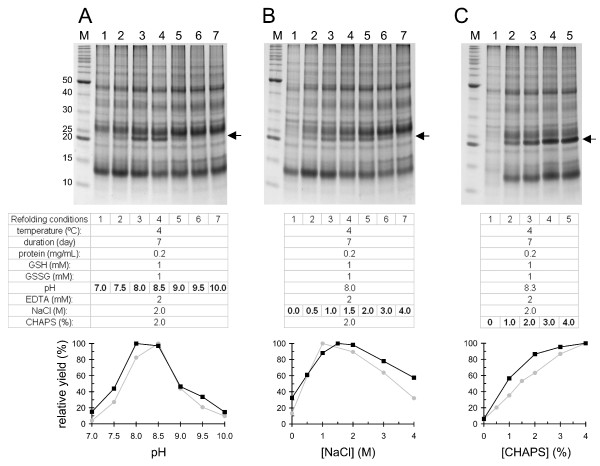
**Effect of host cell contaminants on the refolding of MB109.** Isolated inclusion bodies, without SEC purification, were directly used to set up the refolding at 0.2 mg/mL. Black arrows indicate the refolded functional dimer. **(A)** Refolding at pH 7–10. The densitometry of the functional band in each condition is shown in black squares and lines. Gray squares and lines are the refolding results from Figure 2 for comparison. **(B and C)** Refolding in 0–4 M NaCl **(B)** and 0–4% CHAPS **(C)**.

As shown in the lane 6 of Figure 1B, the isolated inclusion bodies of MB109 contain a visible amount of host cell contaminants on SDS-PAGE. Although these contaminants could be effectively removed by size exclusion chromatography prior to refolding, it would be cost-effective if this purification step were omitted. Therefore, the refolding efficiency was tested in the presence of the host cell contaminants by using the isolated inclusion bodies in the refolding conditions outlined above (variation of pH and salt and CHAPS concentrations). As shown in the upper panels of Figure 3, the functional dimer (black arrows) was refolded in the presence of host cell contaminants. Similar to the size-exclusion purified inclusion bodies, the non-purified inclusion bodies were refolded into functional dimer with an optimal pH of 8.0–8.5 (Figure 3A), an optimal NaCl concentration of 1–2 M (Figure 3B), and a positive correlation with increasing CHAPS detergent concentration, plateauing around 3% (Figure 3C).

### Purification of the refolded MB109

**Figure 4:**
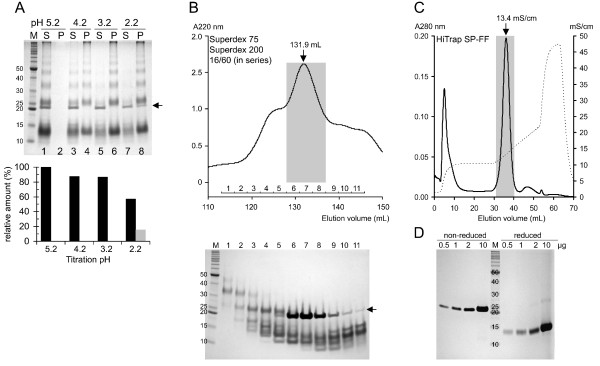
**Purification of the refolded MB109 functional dimer. (A)** Upper panel is a non-reduced SDS-PAGE image showing the aggregation property of the refolded MB109 proteins at pH 5.2, 4.2, 3.2 and 2.2. Black arrow indicates the functional dimer. S: supernatant; P: pellet. Bottom panel is the corresponding densitometry of the functional dimer band in each lane. Black and gray bars represent the relative amounts of functional dimer in supernatants and pellet, respectively. **(B)** Upper panel, size exclusion chromatographic profile of Superdex 75 and 200 16/60 (connected in series) loaded with acidic fractionated supernatant. Gray bar highlights the fractions pooled for next purification step. Bottom panel is non-reduced SDS-PAGE gel image of each corresponding elution fractions. **(C)** Ion exchange chromatographic profile of HiTrap SP-FF (1 mL) loaded with the size-exclusion purified sample. Solid line, absorption of 280 nm; dashed line, conductance. Gray bar highlights the fractions containing the functional dimer. **(D)** Purity analysis of the purified functional dimer on reduced and non-reduced SDS-PAGE gel. The protein was loaded at 0.5, 1, 2, and 10 μg in the absence (left) or presence (right) of 100 mM DTT.

In all of the refolding conditions analyzed above, the denatured MB109 was refolded into not only the functional dimer, but also other variants, including the chemical dimer, monomer and multimer (Figure 2). These variants were considered as contaminants because of their lack of bioactivity, and thus we attempted to separate the variants. To separate the functional dimer from the contaminants, we found that the majority of the contaminants could be effectively separated by precipitation when the pH of the refolding mixture was directly titrated to 3.2. As shown in Figure 4A, the majority of contaminants formed water insoluble aggregates at pH 4.2 and 3.2 and could be removed by centrifugation (lanes 4 and 6, respectively), while the functional dimer remained in the supernatant (lane 3 and 5). At pH 2.2, however, the functional dimer started to form water insoluble aggregates as well (Figure 4A lane 8, black arrow).

After acidic fractionation, small amounts of chemical dimer, monomer and multimer remained in the supernatant (Figure 4A lane 5). To further purify the functional dimer, size exclusion chromatography with Superdex 75 and Superdex 200 connected in series was used. As shown in Figure 4B, the multimer and chemical dimer were eluted at 113–128 mL, before the function dimer was eluted at 128–137 mL (gray box). However, some of the monomeric variants were co-eluted with the functional dimer (Figure 4B, bottom panel, lane 6–8).

To remove the remaining monomeric variants, SP Sepharose Fast Flow cation exchange was found to be effective. As shown in Figure 4C, the majority of the monomeric contaminants were washed out around 10.5 mS/cm and the functional dimer was eluted around 13.4 mS/cm. After this step, the purity of the purified functional dimer was greater than 95%, as determined by non-reduced and reduced SDS-PAGE (Figure 4D). The final yield of the purified MB109 was 7.8 mg per 100 mg of SEC purified inclusion bodies under the optimal refolding conditions.

### Bioactivity of refolded MB109

**Figure 5:**
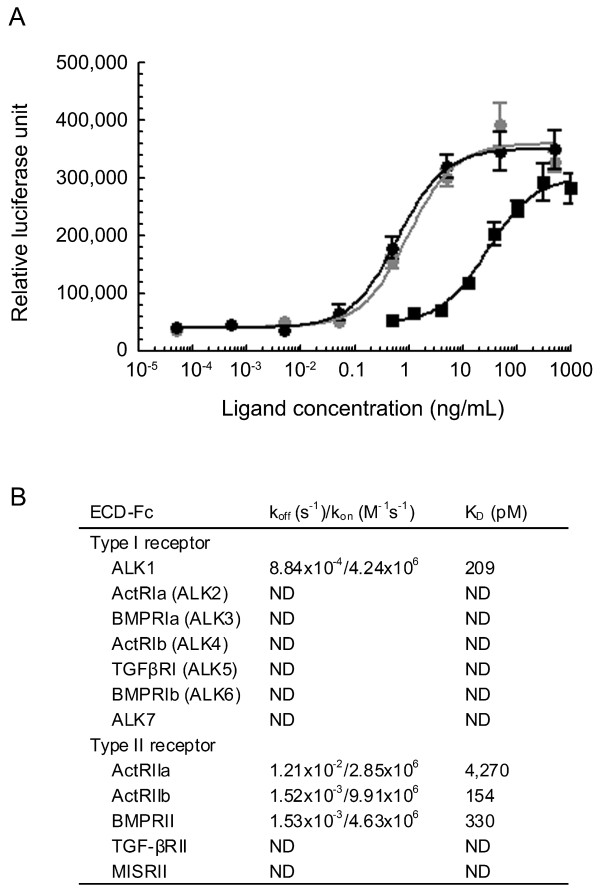
**Activity assays of the purified MB109. (A)** Smad1-depend luciferase reporter assay in mouse myoblast C2C12 cells. Black circles are *E. coli*-derived MB109. Gray circles are CHO-derived hBMP-9. Black squares are *E. Coli*-derived hBMP-2. S.E.M. are shown, n = 3. **(B)** Binding affinities of MB109 to Type I and Type II receptors as determined by surface plasma resonance. ECD-Fc, extracellular domain with C-terminal human IgG_1_ tag. ND, non-detectable (no signal above the background detection was detected in all tested protein concentration ranging from 20 to 0.3 nM).

The bioactivity of the purified MB109 was first tested by examining its ability to stimulate the Smad1 signaling pathway in an established Smad1-dependent luciferase reporter system in mouse myoblast C2C12 cells (Figure 5A). A CHO-derived hBMP-9 and an *E. coli*-derived hBMP-2 were used as positive controls. As shown in Figure 5A, the purified MB109 was able to induce dose-dependent Smad1 signaling response with an EC_50_ of 0.61 ng/mL (25 pM, black circles), which was similar to that induced by CHO-derived hBMP-9 (EC_50_ of 0.92 ng/mL, 38 pM, gray circles). This is about a 43-fold higher response than that induced by *E. coli*-derived hBMP-2 (EC_50_ of 28.1 ng/mL, 1.08 nM, black squares).

To determine receptor binding specificity, purified MB109 was subjected to surface plasmon resonance analysis to determine its binding affinities to immobilized extracellular domains (ECDs) of Type I and Type II receptors, including ALK1, ActRIa (ALK2), ActRIb (ALK4), BMPRIa (ALK3), BMPRIb (ALK6), TGFβRI (ALK5), ALK7, ActRIIa, ActRIIb, BMPRII, TGF-βRII and MISRII. Among these receptor ECDs, MB109 had strong binding affinity only to ALK1, ActRIIb and BMPRII, while the binding to the other receptor ECDs were either very transient (to ActRIIa) or non-detectable (Figure 5B).

## Discussion

The *E. coli* expression system, in combination with a protein refolding technique, is a simple, straightforward, and cost-effective method to produce recombinant TGF-β superfamily ligands. This method takes the advantages of well-established molecular tools and robust protein expression capability of the *E. coli* cells. As compared to the CHO cell expression system, this procedure is particularly suitable to generate a large number of synthetic TGF-β chimeras [18], without the time consuming steps of establishing stably transfected cell lines and screening for individual high expression clones. The *E. coli* expression and refolding method can also be directly incorporated into a mid- or high-throughput activity screen platform to identify novel biologics, similar to the antibody discovery platform [29]. Although many studies on refolding technology exist in the literature, the methods developed for TGF-β superfamily ligands have been mostly limited to BMP-2 and activin A [20-22,30,31]. However, the standard conditions derived from these early studies were not generally applicable to effectively refold other TGF-β superfamily ligands. To overcome this bottleneck step, we performed a comprehensive study on several refolding variables to investigate how each individual refolding variable affects the overall refolding efficiency of BMP-9, which has significant therapeutic interest.

We found that the optimal refolding of MB109 took more than 7 days at 4°C, which is relatively slow compared to the *Drosophila* DPP, hBMP-2 and activin A (3-7 days) [20,30,31]. The refolding efficiency was strongly dependent on pH and the concentrations of NaCl, CHAPS and protein in the refolding solution. The presence of several commonly used denaturants and aggregation suppressors (secondary additives), especially guanidine, had detrimental effect on MB109 refolding. Guanidine was found previously to be advantageous in the refolding of hBMP-2 [22]. The combination of all the tested variables left a very narrow window for MB109 to be refolded with high efficiency. In other words, this narrow window could be easily missed in a refolding screen specially if several variables are varied at the same time or extreme screening conditions are used. The only variable that allowed for refolding in a wide range of conditions was the redox condition. As long as the amount of reducing agent (GSH) was equal to or more than the oxidizing agent (GSSG), MB109 could be refolded with similar efficiency.

Overall, several optimal refolding parameters we identified for MB109 are similar to those conditions used to refold *Drosophila* DPP, hBMP-2, 3, 6, 2/6, 12 and 13 [20,22-26]. This may suggest a common kinetic refolding mechanism of the BMP family ligands [31]. Therefore, perhaps all of the BMP family ligands can be refolded by the common set of chemical components and variables discussed here, only with some variations in their optimal ranges. This concept is particularly valuable for designing a high-throughput refolding platform to refold tens or hundreds of synthetic TGF-β chimeras at the same time. For example, the common set of chemical components and variables could be used in a first-round refolding screen to identify commonly refoldable ligands. Then, other variables, such as detergent, pH, salt or secondary additives, could be varied individually in subsequent screens to find additional refoldable ligands. In addition to the refolding variables, we also showed that the presence of host cell contaminants has no detrimental effect on refolding MB109. A similar result was also observed from a hBMP-2 study [22]. These results suggest that the refolding mechanism of the TGF-β superfamily ligands is fairly robust, such that the denatured polypeptide can effectively form proper disulfide-bonds and dimerize with high efficiency, allowing for a large number of synthetic TGF-β chimera to be refolded in a high-throughput manner without prior chromatographic purification of the inclusion bodies.

It has been shown that different detergents can be used to refold activin A to varied degrees of efficiency, among which TDCA (sodium taurodeoxycholate) was identified to be the most effective [30]. The detergent-like CHES [2-(cyclohexylamino)ethanesulfonic acid] has also been used to refold hBMP-2 [21,22,31] and, as stated above, the CHAPS detergent has been used to refold *Drosophila* DPP, hBMP-2, 3, 6, 2/6, 12 and 13 [20,23-26]. These results indicate that detergent is an indispensable additive to refold TGF-β superfamily ligands; however, the optimal detergent may not be the same for each member, thus necessitating an additional detergent screen. Because the presence of CHAPS allowed for the ability to refold MB109, an additional detergent screen was not performed in this study.

In the purification step, a first attempt had been made to develop a procedure at alkaline pH, as described for *Drosophila* DPP purification [20]. However, the refolded MB109 functional dimer was found to be unstable during the purification process at alkaline pH, presumably due to errors in disulfide bond formation. Because disulfide shuffling can be effectively prevented in acidic conditions, the first step in the purification procedure was designed to address this by directly titrating down the pH of the refolded sample. Interestingly, at acidic pH the refolded functional dimer remained stable, while a majority of other refolded variants (chemical dimer, monomer and multimer) were unstable and formed aggregates (Figure 4A). This biochemical property appears to be common among the TGF-β superfamily ligands, because the refolded variants of hBMP-2 and some of the activin-BMP-2 chimeras [18] can also be precipitated by the same titration method after the refolding step (unpublished observations). After the acidic titration step, small amounts of chemical dimer, monomer and multimer remained (Figure 4A).

To further remove the remaining contaminants, we tested several cation exchange media, such as SP Sepharose Fast Flow, SP Sepharose XL, SP Sepharose High Performance and CM Sepharose Fast Flow (GE Healthcare), but none were able to separate the target from the contaminants in a single chromatographic step. This problem may be due to the fact that the remaining contaminants are chemical dimer, monomer and multimer variants, which have the same amino acid sequence as the functional dimer. The core of each subunit of the functional dimer contains three disulfide bonds, all with nearly the same bonding distances. Because the remaining contaminants have the same amino acid sequence and isoelectronic point, the contaminants may only differ from the functional dimer in the pattern of the formation of these three disulfide bonds, resulting in alternate local conformations.

Due to the lack of success with the cation exchange media to remove the small amount of contaminants after the acidic titration step, we attempted size-exclusion chromatography and found that we were able to separate a portion of the monomeric variants and all of the chemical dimer and multimer from the functional dimer (Figure 4B). The remaining monomeric variants could be subsequently removed effectively with an additional step of cation exchange chromatography. Therefore, the entire purification procedure consisted of two chromatographic steps (size exclusion chromatography followed by cation exchange chromatography). This purification procedure was developed with general applicability in mind, so that it could be applied to purify other TGF-β superfamily ligands as well as synthetic TGF-β superfamily chimeras. Indeed, we found that hBMP-2 and some of the activin-BMP-2 chimeras [18] could be effectively purified by this procedure with minor differences in their chromatographic profiles (unpublished observations). Additionally, when the non-purified inclusion bodies were refolded directly, the host cell contaminants present in the refolded MB109 were removed effectively with the same purification procedure.

Our luciferase reporter assay demonstrated that MB109 is equal to the CHO-derived hBMP-9 in terms of Smad1 signaling capability. The receptor binding analysis by surface plasmon resonance also showed that the *E. coli*-derived MB109 binds specifically to the ALK1 Type I receptor and to the ActRIIb and BMPRII Type II receptors. The receptor binding results are consistent with recent data using CHO-derived hBMP-9 [3]. More recently, we have shown that MB109 enhances brown adipogenesis in human adipose tissue-derived stem cells *in vitro* and suppresses weight gaining in high fat diet-induced obese mice *in vivo*[32]. Our activity data demonstrate that the *E. coli*-derived MB109 has the equal quality and potentials in clinical applications as CHO-derived hBMP-9.

To improve the yield in the *E. coli* expression system and to optimize the refolding platform for large-scale production, several steps in the overall procedure can be further optimized. For example, continuous or pulse refolding, instead of rapid-dilution refolding, may improve the refolding yield, as in the case of hBMP-2 [22]. The cell density of the cell culture, as well as the protein expression, can be obviously improved by using a bioreactor and alternate induction methods over a shaking flask and IPTG induction at 37°C [21], including possible production of nonclassical inclusion bodies, which may bypass the lengthy refolding procedures [33,34]. To simplify the purification step, an affinity column made with the ECDs of the ALK1, ActRIIb or BMPRII receptor may be used to directly capture the MB109 functional dimer after the refolding step to purify the protein in a single chromatographic step. The cost effectiveness of any of these proposed procedures would have to be evaluated.

## Conclusions

This study revealed a simple, straightforward, and cost-effective technique to produce bioactive human BMP-9, as an *E. coli*-derived recombinant protein MB109. Because BMP-9 had never been refolded successfully, and could previously only be produced from the more expensive CHO cell system, MB109 can now be used not only to facilitate BMP-9 related biomedical research, but can also be scaled-up for industrial production. In addition, this study identified the critical variables to refold human BMP-9 with high efficiency. The optimal refolding parameters can serve as a basis for further optimization of the refolding yield utilizing alternate refolding techniques. In conclusion, these findings add to the current understanding of how to improve the refolding efficiency of TGF-β superfamily ligands. This is particularly valuable when a synthetic TGF-β superfamily library, containing tens or hundreds of chimeras, could to be produced simultaneously for a rapid activity screen.

## Methods

### Molecular biology and E. coli cell culture

The gene of the mature domain of human BMP-9 (Ser320 – Arg429), containing a 5′ start codon and a 3′ stop codon, was synthesized by Genscript (NJ, USA). The codon usage was optimized for maximal *E. coli* expression. The synthetic gene was ligated into a pET21a vector (Novagen, USA) between the NdeI and XhoI restriction enzyme sites to make the expression plasmid. The sequence was confirmed by DNA sequencing after cloning.

For protein expression, the plasmid was transformed into BL21 *E. coli* cells by heat shock. The transformants were selected on an LB-broth agar plate, containing 100 μg/mL ampicillin (LBamp). A single colony was inoculated in 10 mL LBamp medium and incubated aerobically at 37°C for 16 hours. The overnight culture was diluted 200-fold into 1 L fresh LBamp medium and incubated aerobically in a shaking incubator at 37°C. When the cell density reached OD_600_ ~0.4, 0.5 mM IPTG (isopropyl β-D-1-thiogalactopyranoside) was added into the cell culture to induce the protein expression. After 20 hours of expression at 37°C, the cells were harvested by centrifugation.

### Inclusion body isolation and chromatographic purification of denatured MB109

To isolate inclusion bodies, the cells were resuspended in deionized water in the presence of 1 mM phenylmethylsulfonyl fluoride (PMSF) and lysed by passing through a Nano DeBEE microfluidizer. The inclusion bodies in the cell lysate were precipitated by centrifugation, and washed three times by repeated resuspension and centrifugation. The washed inclusion bodies were dissolved in an 8 M urea buffer solution in the presence of dithiothreitol (DTT) and stored at -80°C to be used for setting up refolding directly or for further chromatographic purification.

The gel filtration chromatography was performed with an Akta Prime plus FPLC. HiLoad 16/60 Superdex™ 75 and 200 columns (GE Healthcare) were used to elute the protein. The fractions containing monomeric MB109 were pooled, concentrated by Vivaspin20 concentrators (Sartorius AG, Germany) and stored at -80°C to be later used for refolding. The protein concentration was determined by using the Bio-Rad Protein Assay Kit (Bio-Rad Laboratories, USA).

### Chemical refolding and purification of refolded MB109

The small-scale refolding screen analyses were performed with a final volume of 1–5 mL. For rapid dilution, the purified monomeric MB109 was diluted directly into cold refolding solutions, mixed rapidly by vortexing and incubated at 4°C. To visualize the progress of refolding, visible protein aggregates in the sample solutions were spun down first and the supernatants were diluted in an acidic buffer solution to stop disulfide reshuffling or redox reaction and concentrated to run on non-reduced 12% SDS-PAGE. The densitometry of the functional dimer bands on the SDS-PAGE gel images was analyzed by using the GeneTools image analysis software (Synoptics, UK). To purify the refolded MB109 functional dimer, the pH of the refolded sample was titrated directly to 3.2 and subjected to gel filtration chromatography by HiLoad 16/60 Superdex™ 75 and 200 columns (Figure 4B). For cation exchange chromatography, 1-mL HiTrap SP Sepharose Fast Flow column (GE Healthcare) was used with the elution salt gradient ranging from 0 to 1 M NaCl (Figure 4C).

### Smad1-dependent luciferase reporter assay

C2C12 cells were purchased from American Type Culture Collection and were cultured in Dulbecco’s modified eagle’s medium (DMEM) containing 100 U/mL penicillin, 0.1 μM streptomycin and 10% FBS. The cells were incubated at 37°C under a humidified condition of 5% CO_2_ and routinely subcultured using trypsine-EDTA when the cell density reached around 80% confluence. Smad1-dependent luciferase report assays were performed as previously described [18,24,35]. In short, cultured C2C12 cells were trypsinized, washed once with PBS, resuspended in OptiMEM (Invitrogen, USA) plus 0.1% FBS and seeded in 96-well plates at 15,000 cells per 80 μL per well. Immediately after seeding, cells were transfected with the -1147Id1-luciferase plasmid [35], a Smad1 expression plasmid and a beta-galactosidase expression plasmid using Fugene6 (Promega, USA) according to the manufacturer’s instruction. After 24 hours of growth, 10 μL of series diluted ligands in OptiMEM medium were added into the cell culture in triplication. After 16 hours of treatment, the cells were washed once with PBS and lysed for measuring luciferase and beta-galactosidase activities using a Mithras LB 940 microplate reader (Berthold Technologies, Germany). The luciferase activity in each well was normalized with the beta-galactosidase activity, and the data were analyzed by using Prism 5 software (GraphPad Software, Inc., USA). The CHO-derived hBMP-9 was purchased from R&D Systems (Minneapolis, USA). The *E. coli*-derived hBMP-2 was purchased from joint Protein Central (http://www.jointproteincentral.com, Incheon, Korea).

### Surface plasmon resonance analysis

The proteins of the extracellular domain (ECD) of Type I and Type II receptors were purchased from R&D Systems (Minneapolis, USA) as C-terminal Fc-tagged chimeras. The binding affinities between MB109 and the ECDs were measured using a BIAcore 3000 (GE Healthcare, USA) at 25°C following a protocol described previously with minor modification [18,26]. In brief, the ECD-Fc proteins were prepared in a pH 4.0 solution (10 mM Na-acetate, pH 4.0) and were captured on the flow cell 2, 3 and 4 of the CM5 sensor chip at a flow rate of 10 μL/min until the density reached ~1,000 response units. Flow cell 1 was left blank for background subtraction. For kinetic analysis, purified MB109 was prepared in a series of 2-fold dilution, from 20 to 0.3 nM, in an assay solution (10 mM HEPES, pH 7.4, 150 mM NaCl, 3.4 mM EDTA and 0.005% Tween-20) and injected over the flow cells at a flow rate of 50 μL/ml. All tests were performed using the set of 2-fold diluted protein plus the assay solution as a blank. The data fitting was performed using a minimum of five protein concentrations and a global 1:1 Langmuir binding with mass transfer model.

## Competing interests

A patent application has been filed.

## Authors’ contributions

MMK conceived and coordinated the study, and participated in its design. PHN carried out the surface plasmon resonance analysis. YJ carried out the luciferase reporter assay. SK and SY performed molecular cloning, cell culture, refolding analyses and protein purification. MMK and SC interpreted and evaluated the data and wrote the manuscript. All authors read and approved the final manuscript.

## References

[B1] Brown MA Zhao QH Baker KA Naik C Chen C Pukac L Singh M Tsareva T Parice Y Mahoney A Crystal structure of BMP-9 and functional interactions with pro-region and receptors J Biol Chem 2005 280 26 25111 25118 10.1074/jbc.M503328200 15851468

[B2] David L Mallet C Mazerbourg S Feige JJ Bailly S Identification of BMP9 and BMP10 as functional activators of the orphan activin receptor-like kinase 1 (ALK1) in endothelial cells Blood 2007 109 5 1953 1961 10.1182/blood-2006-07-034124 17068149

[B3] Townson SA Martinez-Hackert E Greppi C Lowden P Sako D Liu J Ucran JA Liharska K Underwood KW Seehra J Specificity and structure of a high affinity activin receptor-like kinase 1 (ALK1) signaling complex J Biol Chem 2012 287 33 27313 27325 10.1074/jbc.M112.377960 22718755 PMC3431715

[B4] Ye L Kynaston H Jiang WG Bone morphogenetic protein-9 induces apoptosis in prostate cancer cells, the role of prostate apoptosis response-4 Mol Cancer Res 2008 6 10 1594 1606 10.1158/1541-7786.MCR-08-0171 18922975

[B5] Herrera B van Dinther M ten Dijke P Inman GJ Autocrine bone morphogenetic protein-9 signals through activin receptor-like kinase-2/Smad1/Smad4 to promote ovarian cancer cell proliferation Cancer Res 2009 69 24 9254 9262 10.1158/0008-5472.CAN-09-2912 19996292 PMC2892305

[B6] Li Q Gu X Weng H Ghafoory S Liu Y Feng T Dzieran J Li L Ilkavets I Kruithof- de Julio M Bone morphogenetic protein-9 induces epithelial to mesenchymal transition in hepatocellular carcinoma cells Cancer Sci 2013 104 3 398 408 10.1111/cas.12093 23281849 PMC7657113

[B7] Scharpfenecker M van Dinther M Liu Z van Bezooijen RL Zhao QH Pukac L Lowik C ten Dijke P BMP-9 signals via ALK1 and inhibits bFGF-induced endothelial cell proliferation and VEGF-stimulated angiogenesis J Cell Sci 2007 120 6 964 972 10.1242/jcs.002949 17311849

[B8] Suzuki Y Ohga N Morishita Y Hida K Miyazono K Watabe T BMP-9 induces proliferation of multiple types of endothelial cells in vitro and in vivo J Cell Sci 2010 123 10 1684 1692 10.1242/jcs.061556 20406889

[B9] Cheng HW Jiang W Phillips FM Haydon RC Peng Y Zhou L Luu HH An NL Breyer B Vanichakarn P Osteogenic activity of the fourteen types of human bone morphogenetic proteins (BMPs) J Bone Joint Surg Am 2003 85A 8 1544 1552 12925636 10.2106/00004623-200308000-00017

[B10] Kang Q Sun MH Cheng H Peng Y Montag AG Deyrup AT Jiang W Luu HH Luo J Szatkowski JP Characterization of the distinct orthotopic bone-forming activity of 14 BMPs using recombinant adenovirus-mediated gene delivery Gene Ther 2004 11 17 1312 1320 10.1038/sj.gt.3302298 15269709

[B11] Majumdar MK Wang E Morris EA BMP-2 and BMP-9 promote chondrogenic differentiation of human multipotential mesenchymal cells and overcome the inhibitory effect of IL-1 J Cell Physiol 2001 189 3 275 284 10.1002/jcp.10025 11748585

[B12] Blunk T Sieminski AL Appel B Croft C Courter DL Chieh JJ Goepferich A Khurana JS Gooch KJ Bone morphogenetic protein 9: a potent modulator of cartilage development in vitro Growth Factors 2003 21 2 71 77 10.1080/0897719031000148822 14626354

[B13] Luther G Wagner ER Zhu GH Kang Q Luo Q Lamplot J Bi Y Luo XJ Luo JY Teven C BMP-9 induced osteogenic differentiation of mesenchymal stem cells: molecular mechanism and therapeutic potential Curr Gene Ther 2011 11 3 229 240 10.2174/156652311795684777 21453282

[B14] Lopez-Coviella I Berse B Krauss R Thies RS Blusztajn JK Induction and maintenance of the neuronal cholinergic phenotype in the central nervous system by BMP-9 Science 2000 289 5477 313 316 10.1126/science.289.5477.313 10894782

[B15] Chen C Grzegorzewski KJ Barash S Zhao QH Schneider H Singh M Pukac L Bell AC Duan R Coleman T An integrated functional genomics screening program reveals a role for BMP-9 in glucose homeostasis Nat Biotechnol 2003 21 3 294 301 10.1038/nbt795 12598908

[B16] Rosen VA Wozney JM Celeste AJ Thies RS Song JR BMP-9 compositions 2001 Patent Number US6287816 B1

[B17] Berasi S Brown CT Cain MJ Calabro V Juo ZS Martinez RV Seeherman H Wozney J Designer osteogenic proteins 2012 Patent Number US20120046227 A1

[B18] Allendorph GP Read JD Kawakami Y Kelber JA Isaacs MJ Choe S Designer TGF beta superfamily ligands with diversified functionality PLoS ONE 2011 6 11 e26402 10.1371/journal.pone.0026402 22073163 PMC3208551

[B19] Ruppert R Hoffmann E Sebald W Human bone morphogenetic protein 2 contains a heparin-binding site which modifies its biological activity Eur J Biochem 1996 237 1 295 302 10.1111/j.1432-1033.1996.0295n.x 8620887

[B20] Groppe J Rumpel K Economides AN Stahl N Sebald W Affolter M Biochemical and biophysical characterization of refolded Drosophila DPP, a homolog of bone morphogenetic proteins 2 and 4 J Biol Chem 1998 273 44 29052 29065 10.1074/jbc.273.44.29052 9786911

[B21] Vallejo LF Brokelmann M Marten S Trappe S Cabrera-Crespo J Hoffmann A Gross G Weich HA Rinas U Renaturation and purification of bone morphogenetic protein-2 produced as inclusion bodies in high-cell-density cultures of recombinant Escherichia coli J Biotechnol 2002 94 2 185 194 10.1016/S0168-1656(01)00425-4 11796171

[B22] Vallejo LF Rinas U Optimized procedure for renaturation of recombinant human bone morphogenetic protein-2 at high protein concentration Biotechnol Bioeng 2004 85 6 601 609 10.1002/bit.10906 14966801

[B23] Allendorph GP Isaacs MJ Kawakami Y Belmonte JCI Choe S BMP-3 and BMP-6 structures illuminate the nature of binding specificity with receptors Biochemistry 2007 46 43 12238 12247 10.1021/bi700907k 17924656

[B24] Isaacs MJ Kawakami Y Allendorph GP Yoon B-H Belmonte JCI Choe S Bone morphogenetic protein-2 and -6 heterodimer illustrates the nature of ligand-receptor assembly Mol Endocrinol 2010 24 7 1469 1477 10.1210/me.2009-0496 20484413 PMC2903903

[B25] Berasi SP Varadarajan U Archambault J Cain M Souza TA Abouzeid A Li J Brown CT Dorner AJ Seeherman HJ Divergent activities of osteogenic BMP2, and tenogenic BMP12 and BMP13 independent of receptor binding affinities Growth Factors 2011 29 4 128 139 10.3109/08977194.2011.593178 21702718 PMC3154542

[B26] Allendorph GP Vale WW Choe S Structure of the ternary signaling complex of a TGF-beta superfamily member Proc Natl Acad Sci U S A 2006 103 20 7643 7648 10.1073/pnas.0602558103 16672363 PMC1456805

[B27] Clark EDB Schwarz E Rudolph R Inhibition of aggregation side reactions during in vitro protein folding Methods Enzymol 1999 309 217 236 10507027 10.1016/s0076-6879(99)09017-5

[B28] Meng FG Park YD Zhou HM Role of proline, glycerol, and heparin as protein folding aids during refolding of rabbit muscle creatine kinase Int J Biochem Cell Biol 2001 33 7 701 709 10.1016/S1357-2725(01)00048-6 11390278

[B29] Cariuk P Gardener M Vaughan T Evolution of biologics screening technologies Pharmaceuticals 2013 6 5 681 688 10.3390/ph6050681 24276173 PMC3817722

[B30] Ejima D Ono K Tsumoto K Arakawa T Eto Y A novel “reverse screening” to identify refolding additives for activin-A Protein Expr Purif 2006 47 1 45 51 10.1016/j.pep.2005.08.013 16226036

[B31] Vallejo LF Rinas U Folding and dimerization kinetics of bone morphogenetic protein-2, a member of the transforming growth factor-β family FEBS J 2013 280 83 92 10.1111/febs.12051 23122408

[B32] Kuo MM-C Kim S Tseng C-Y Jeon Y-H Choe S Lee DK BMP-9 as a potent brown adipogenic inducer with anti-obesity capacity Biomaterials 2014 35 10 3172 3179 10.1016/j.biomaterials.2013.12.063 24439409

[B33] Jevševar S Gaberc-Porekar V Fonda I Podobnik B Grdadolnik J Menart V Production of nonclassical inclusion bodies from which correctly folded protein can be extracted Biotechnol Prog 2005 21 2 632 639 15801811 10.1021/bp0497839

[B34] Peternel Š Grdadolnik J Gaberc-Porekar V Komel R Engineering inclusion bodies for non denaturing extraction of functional proteins Microb Cell Factories 2008 7 34 1 9 10.1186/1475-2859-7-34 PMC2630956 19046444

[B35] Nakashima K Takizawa T Ochiai W Yanagisawa M Hisatsune T Nakafuku M Miyazono K Kishimoto T Kageyama R Taga T BMP2-mediated alteration in the developmental pathway of fetal mouse brain cells from neurogenesis to astrocytogenesis Proc Natl Acad Sci 2001 98 10 5868 5873 10.1073/pnas.101109698 11331769 PMC33305

